# Ursolic Acid Inhibits Epithelial-Mesenchymal Transition through the Axl/NF-*κ*B Pathway in Gastric Cancer Cells

**DOI:** 10.1155/2019/2474805

**Published:** 2019-06-09

**Authors:** Jinxia Li, Chunyan Dai, Li Shen

**Affiliations:** ^1^Hunan University of Chinese Medicine, Changsha 410208, China; ^2^Zhejiang Key Laboratory of Gastro-Intestinal Pathophysiology, Zhejiang Hospital of Traditional Chinese Medicine, Zhejiang Chinese Medicine University, Hangzhou 310006, China; ^3^Institute of Basic Theory of TCM, China Academy of Chinese Medical Sciences, Beijing 100700, China

## Abstract

**Background:**

Ursolic acid (UA) is an antitumor component derived from Chinese herbal medicine; this study is to observe the effects of UA on epithelial-mesenchymal transition (EMT) in gastric cancer.

**Methods:**

(1)* In vitro* experiments: 25*μ*mol/L and 50*μ*mol/L UA were applied to BGC-823, AGS, MGC-803, and HGC-27 cells; MTT staining, Transwell assay, and flow cytometry were used to assess cell proliferation, cell migration, and apoptosis, respectively. Western blot was performed to detect the expressions of N-Cadherin, Vimentin, Snail, Twist, Axl, p-Axl, IKK, p-IKK, NF-*κ*B, and p-NF-*κ*B. (2)* In vivo* experiments: Ten BALB/c-nu mice were used to establish gastric cancer xenograft model. Five were orally given UA for 4 weeks and five were given normal saline. Expressions of N-Cadherin and Snail were examined by immunohistochemical assay; expressions of N-Cadherin, Snail, Twist, Axl, p-Axl, IKK, and p-IKK were detected by Western blot.

**Results:**

(1) UA inhibited cell proliferation in BGC-823 and HGC-27 cells in dose-dependent manners. (2) UA inhibited cell migration in BGC-823, AGS, and MGC-803 cells while inducing apoptosis in BGC-823 cells. (3) UA significantly decreased the expressions of N-Cadherin, Vimentin, Snail, Twist p-Axl, p-IKK*α*/*β*, and p-NF-*κ*B in BGC-823 and MGC-803 cells. (4) UA distinctly decreased the expressions of N-Cadherin, Snail, p-Axl, and p-IKK*α*/*β* in gastric cancer xenograft model rats.

**Conclusion:**

UA can effectively inhibit the proliferation and migration and induce apoptosis of gastric cancer cells. The antitumor effect of UA is conducted by EMT inhibition, which may be associated with the regulation of Axl/NF-*κ*B signaling pathway.

## 1. Background

Gastric carcinoma (GC) is the fourth most common malignant tumor throughout world, and it is the second leading cause of cancer mortality [1]. In China, the incidence of GC ranks third among all malignant tumors; there are 380,000 new cases annually [2]. The prognosis of GC is closely related to early detection, invasion, and metastasis which account for more than 90% of the causes of death [3]. Therefore, prevention of tumor invasion and metastasis may improve the early detection and prognosis of GC.

The epithelial-mesenchymal transition (EMT) is a process through which epithelial cells are converted into mesenchymal cells; it is characterized as the loss of cell-cell adhesion and cell polarity, the acquisition of migratory and invasive properties, and the improvement of resistance to apoptosis and degradation of extracellular matrix [4]. Therefore, aberrant activation of EMT plays a crucial role in the genesis, invasion, and metastasis of various tumors, including GC [5].

**Figure 1:**
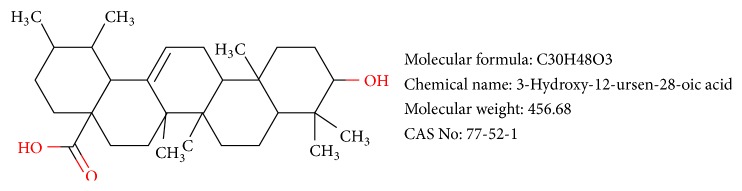
Structural formula of ursolic acid.

Ursolic acid (UA) is an antitumor component derived from Chinese herbal medicine. The structural formula is shown in Figure 1. It could inhibit cell proliferation and induce apoptosis in a variety of tumor cells [6]. Previous studies demonstrated that UA can induce apoptosis and inhibit proliferation of GC [7–9]; hence we hypothesize that UA can prevent GC invasion and metastasis by inhibiting EMT.

In this study, we conducted both* in vivo* and* in vitro* experiments to observe the effects of UA on EMT in GC. In* in vitro* experiment, BGC-823, AGS, MGC-803, NCI-N87, and HGC-27 cells were used; MGC-803 represents high differentiation stage of GC, BGC–823 and AGS represent poorly differentiated GC, HGC-27 represents undifferentiated GC, and NCI-N87 represents fore-GC. In* in vivo *experiment, gastric cancer xenograft mice model was used, since it well simulates the pathogenesis and pathological change of human gastric cancer.

## 2. Methods

### 2.1. *In Vitro* Experiments

#### 2.1.1. Cell Culture

Gastric cancer cell lines BGC-823, HGC-27, AGS, MGC-803, and NCI-N87 were from Central Research Center, Zhejiang Province Hospital of Traditional Chinese Medicine.

After cell resuscitation, cells were inoculated in a 25cm^2^ cell culture flask with a density of 2×10^6^/mL. Cells were maintained in RPMI-1640 medium with 10% calf serum and grew at 37°C in a CO_2_ incubator. Cells in logarithmic growth phase were collected for following experiments.

#### 2.1.2. Cell Proliferation Assay by MTT Staining

250*μ*L of BGC-823 and HGC-27 (3×10^5^/mL) were added to each well in the 96-well plate. UA was added to the final concentration of 6.25*μ*mol/L, 12.5*μ*mol/L, 25*μ*mol/L, 50*μ*mol/L, and 100*μ*mol/L, respectively. Dimethyl sulfoxide and culture medium were added as control. 10*μ*L Cell Counting Kit-8 was added, plates were lucifuge incubated in 37°C for 1h, and the absorbance values were examined. The inhibiting proliferation rates were calculated using the following equation: Inhibiting proliferation rate (%) = (a value of control group - a value of treated group)/ a value of control group×100%.

#### 2.1.3. Cell Migration Assay by Transwell

100*μ*L of AGS, HGC-27, MGC-803, and NCI-N87 were inoculated in the upper chambers, and the cell density was adjusted to 1.5×10^4^/mL and 2×10^4^/mL, respectively. 500*μ*L of culture medium with 10% calf serum was added in the lower chambers; the chambers were incubated at 37°C, 5% CO_2_ for 24h, then rinsed with phosphate buffer saline, fixed with 4% formalin for 20min, and crystal violet stained for 20min. Cotton swabs were used to remove the cells that are unable to penetrate the membrane surface. Photograph under 200× magnification and count the number of migrated cells.

According to the above experimental results, 10*μ*L of UA was added to AGS, MGC-803, and BGC-823 cells with the final concentration of 25*μ*mol/L and 50*μ*mol/L, respectively; after incubation for 24h, the Transwell assay was performed again according to the above steps.

#### 2.1.4. Cell Apoptosis Assay by Flow Cytometry

BGC-823, MGC-803, and AGS cells (1.5×10^5^/mL) were treated with UA at concentration of 50*μ*mol/L for 24h. Cells were collected after centrifugation, 300*μ*L 1×Binding Buffer was added to resuspend the cells, 5*μ*L Annexin V-FITC Early Apoptosis Detection Kit (bioSmile), and 5*μ*L propidine iodide was added and lucifuge incubated for 15min and 5min under room temperature, respectively. Then 200*μ*L 1×Binding Buffer was replenished; flow cytometry was used for apoptosis analysis.

#### 2.1.5. Western Blot Analysis

BGC-823, MGC-803, and AGS cells (2×10^5^/mL) were treated with 25*μ*mol/L and 50*μ*mol/L UA; expressions of N-Cadherin, Vimentin, Snail, Twist, Axl, p-Axl, IKK*α*/*β*, p-IKK*α*/*β*, NF-*κ*B, and p-NF-*κ*B were examined by Western blot. Cells were treated with RIPA Lysis Buffer (1% deoxycholate, 0.1% sodium dodecyl sulfate). Concentrations of proteins were determined by bicinchoninic acid assay. Proteins were separated by sodium dodecyl sulfate-polyacrylamide gel electrophoresis and incubated with proper primary and secondary antibodies. Then the proteins were transferred to polyvinylidene fluoride membrane, and the integrated option density was detected by far infrared scanner (ChemiDocTM Touch Imaging System, Bio-Rad) and Image J software. The relative expressions of proteins were calculated by using *β*-actin as an internal control.

### 2.2. *In Vivo* Experiments

#### 2.2.1. Animals

Fourteen male BALB/c-nu mice weighing 17~20g were purchased and fed in animal experiment center of Zhejiang University of Traditional Chinese Medicine. Animals were housed in a specific pathogen free grade environment and fed with normal water and food. All procedures were under the approval of the Laboratory Animal Management and Welfare Ethical Review Committee of Zhejiang University of Traditional Chinese Medicine (Registration No. ZSLL-2017-1015).

#### 2.2.2. Mouse Model and Grouping

Establishment of subcutaneous xenograft tumors: 5×10^7^/mL of BGC-823 cell suspension was injected into the axillary skin of 4 BALB/c-nu mice; condition was observed every 3 days. After 2 weeks, mice were sacrificed by carbon dioxide euthanasia when the tumor grew to a diameter of 1cm. The underarm skin was routinely disinfected; the tumor tissues were peeled and cut into small pieces about 1mm^3^.

Establishment of GC xenograft model: Ten nude mice were anaesthetized by intraperitoneal injection of pentobarbital sodium peritoneal (50mg/kg); a 0.2cm long incision was cut in the right anterior armpit. The tumor was then implanted into the incision and a drop of OB glue was put to bind the incision. The condition of mice was observed every day to determine the tumorigenesis. At 72h after successful modeling, mice were randomly divided into the model group and UA group; each has 5 mice. UA group was orally given UA 50mg/kg/d, and corresponding saline was given to the model group for four weeks. Under anesthesia by pentobarbital sodium, the tumor tissues were removed, and mice were sacrificed by carbon dioxide euthanasia.

#### 2.2.3. Immunohistochemical Analysis

Expressions of N-Cadherin and Snail in mouse GC xenograft model were examined by immunohistochemistry staining. Sections were routinely dewaxed and hydrated. After the heat-mediated antigen retrieval, the sections were blocked with 10% goat serum at 37°C for 1 h. Then the sections were incubated with primary antibody (1:800) at 4°C overnight. Polink-1 horse radish peroxidase-diaminobenzidine 3 detection system was used to incubate the sections at 37°C for 1 h, followed by diaminobenzidine 3 coloration and haematoxylin restaining. The integral optical density was analyzed by Image Pro Plus 6.0 software.

#### 2.2.4. Western Blot Analysis

Expressions of N-Cadherin, Snail, Axl, p-Axl, IKK*α*/*β*, and p-IKK*α*/*β* in mouse GC xenograft model were determined by Western blot. Tumor tissues were homogenized, and the following operations were as same as above.

### 2.3. Statistical Analysis

Statistical analysis was performed by using SPSS19.0 software. Data were presented as mean ± SD. One-way ANOVA was used for comparison between groups. Least-significant difference test was applied when the variance was equal; otherwise Tamhane's T2 test was performed. A value of P < 0.05 was considered to be statistically significant.

## 3. Results

### 3.1. UA Inhibited Cell Proliferation and Induced Apoptosis in Gastric Cancer Cells

**Figure 2:**
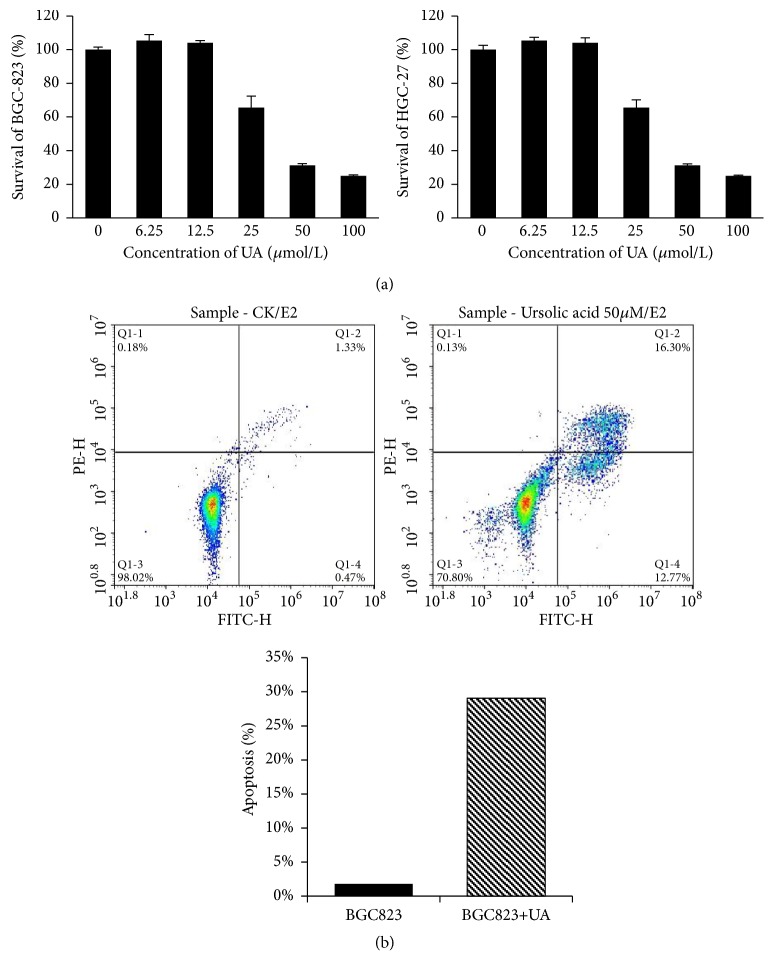
*UA suppressed cell growth and induced apoptosis in gastric cancer cells.* Note: Q1-1: necrotic cells; Q1-2: advanced apoptotic cells; Q1-3: normal cells; Q1-4: early apoptosis cells. Apoptosis rate%= early apoptosis rate% + advanced apoptosis rate%.

Proliferation of BGC-823 and HGC-27 cells was examined to assess the inhibitory effect on cell growth of UA. As shown in Figure 2(a), compared with control group, UA (25-100*μ*mol/L) inhibited cell proliferation in dose-dependent manners both in BGC-823 and in HGC-27 cells.

Apoptosis in BGC-82, MGC-803, and AGS cells was assessed by flow cytometry. As shown in Figure 2(b) the apoptosis rate of normal BCG-823 cells was 1.80%; after 50*μ*mol/L UA was added, the apoptosis rate increased to 29.07%, which indicates that UA can significantly induce apoptosis in BGC-823 cells. UA damaged MGC-803 and AGS cells too much to obtain corresponding result.

### 3.2. Cell Migration Capacity of AGS, HGC-27, MGC-803, and NCI-N87 Cells

**Figure 3:**
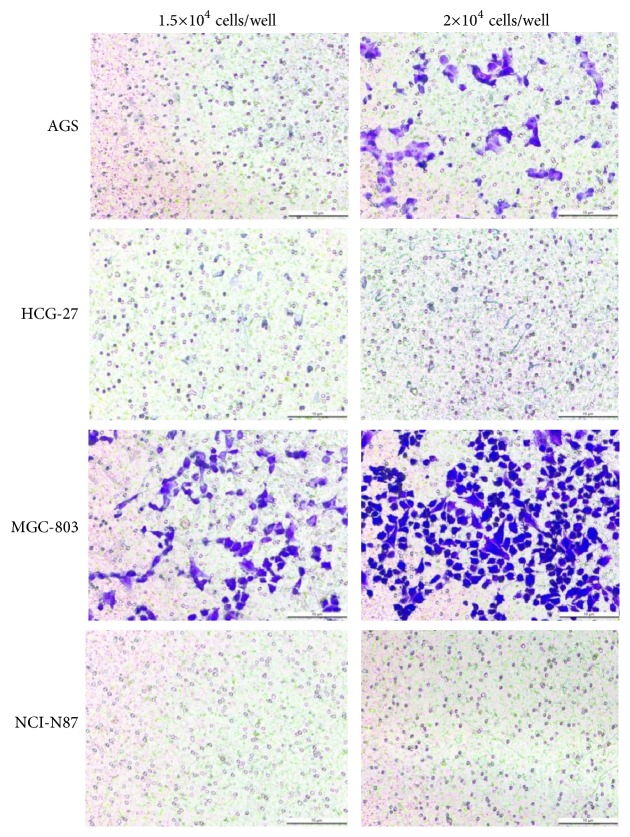
Cell migration capacity of AGS, HGC-27, MGC-803, and NCI-N87 cells.

The cell migration capacity was analyzed by Transwell assay. As shown in Figure 3, AGS and MGC-803 cells migrated significantly at the density of 1.5~2×10^4^ cells/well, while HGC-27 and NCI-N87 cells showed no distinct migration even at the density of 2×10^4^ cells/well. Our previous study showed that BGC-823 cells migrated significantly under the condition of 5×10^4^ cells/well. Therefore, AGS, MGC-803, and BGC-823 cells were selected for further experiments.

### 3.3. UA Suppressed Cell Migration and EMT Related Protein Expressions in MGC-803 and BGC-823 Cells

**Figure 4:**
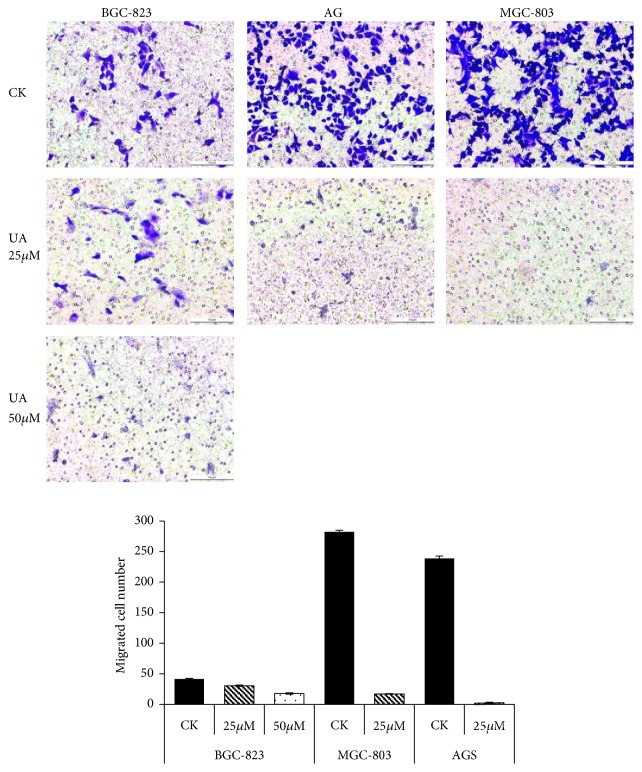
*UA suppressed cell migration in BGC-823, MGC-803, and AGS Cells.* Note: CK: cell lines without treatment; 25*μ*M: CK+25*μ*mol/L UA; 50*μ*M: CK+50*μ*mol/L UA.

As shown in Figure 4, UA (25-50*μ*mol/L) significantly inhibited BGC-823 cell migration in dose-dependent manners. 25*μ*mol/L UA had obvious inhibitory effect on the migration of AGS and MGC-803 cells, but 50*μ*mol/L UA damaged the two groups of cells, leading to the inability of subsequent experiments.

**Figure 5:**
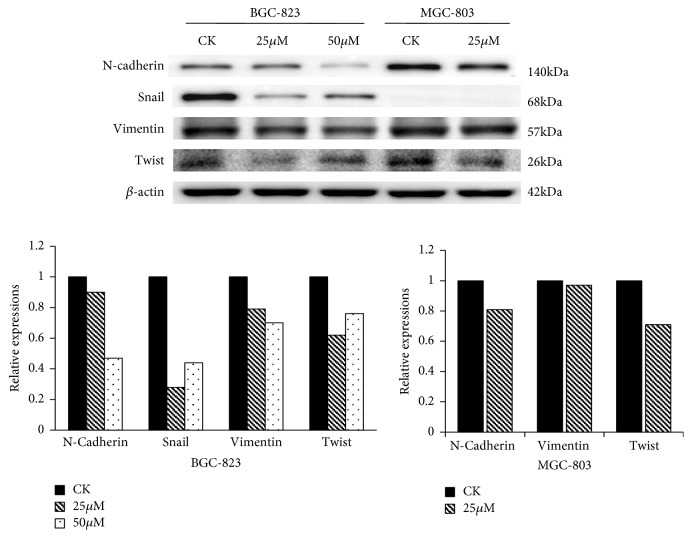
*UA inhibited EMT related protein expressions in MGC-803 and BGC-823 cells.* Note: CK: cell lines without treatment; 25*μ*M: CK+25*μ*mol/L UA; 50*μ*M: CK+50*μ*mol/L UA.

The expressions of EMT related proteins were examined by Western blot. As shown in Figure 5, both 25*μ*mol/L and 50*μ*mol/L UA decreased the levels of N-Cadherin, Snail, Vimentin, and Twist in BGC-823 cells. 25*μ*mol/L UA decreased the levels of N-Cadherin, Vimentin, and Twist in MGC-803 cells. 50*μ*mol/L UA damaged the cells too much to obtain corresponding result in MGC-803 cells.

### 3.4. UA Targeted Axl/NF-*κ*B Pathway in BGC-823 Cells

**Figure 6:**
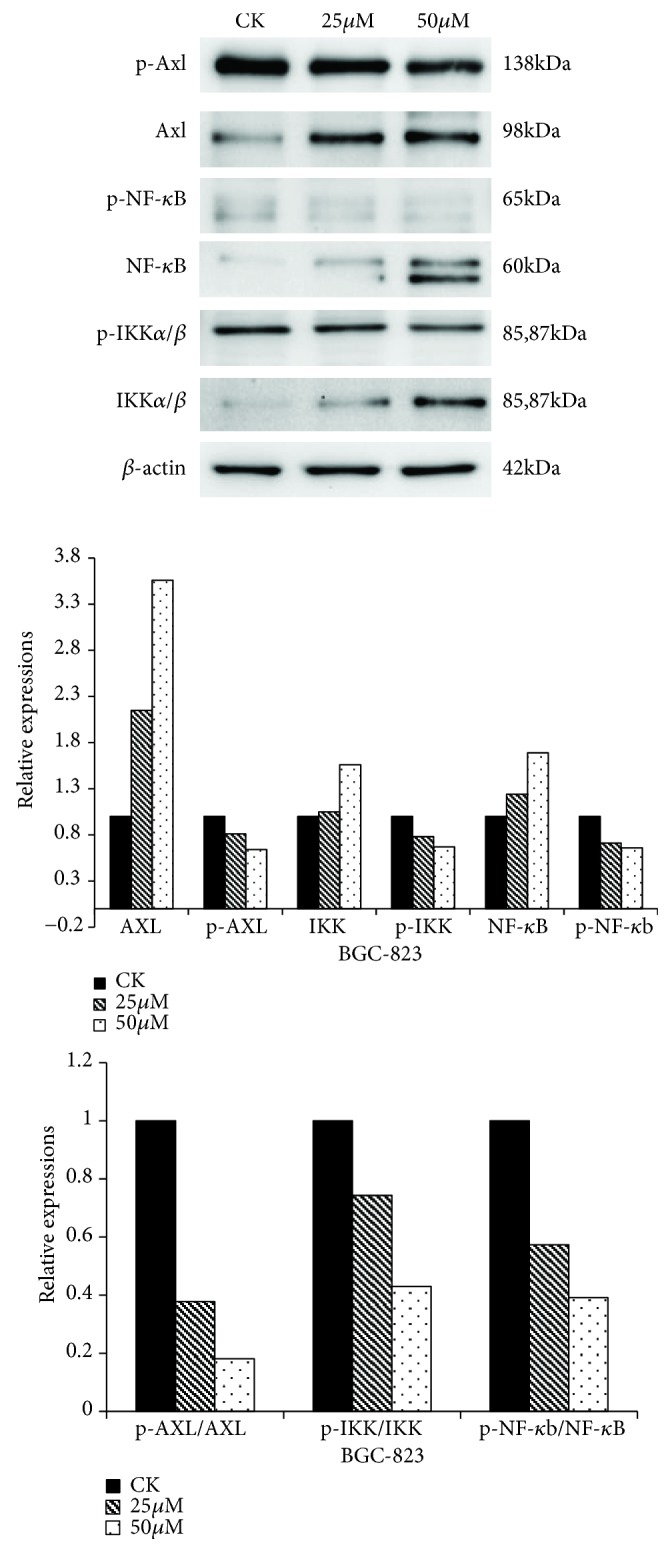
*UA targeted Axl/NF-κB pathway in BGC-823 cells.* Note: CK: cell lines without treatment; 25*μ*M: CK+25*μ*mol/L UA; 50*μ*M: CK+50*μ*mol/L UA.

To investigate the mechanism on how UA regulates EMT, the expressions of key proteins in Axl/NF-*κ*B pathway were examined by Western blot. As shown in Figure 6, UA decreased the levels of p-Axl, p-NF-*κ*B, and p-IKK*α*/*β*, while increasing the levels of Axl, NF-*κ*B, and IKK*α*/*β*, and the values of p-Axl/Axl, p-IKK/IKK, and p-NF-*κ*B/NF-*κ*B were decreased. These results indicate that phosphorylations of Axl, IKK, and NF-*κ*B have been inhibited by UA in BGC-823 cells.

### 3.5. UA Inhibited the Expressions of EMT Related Proteins in Mouse GC Xenograft Model

**Figure 7:**
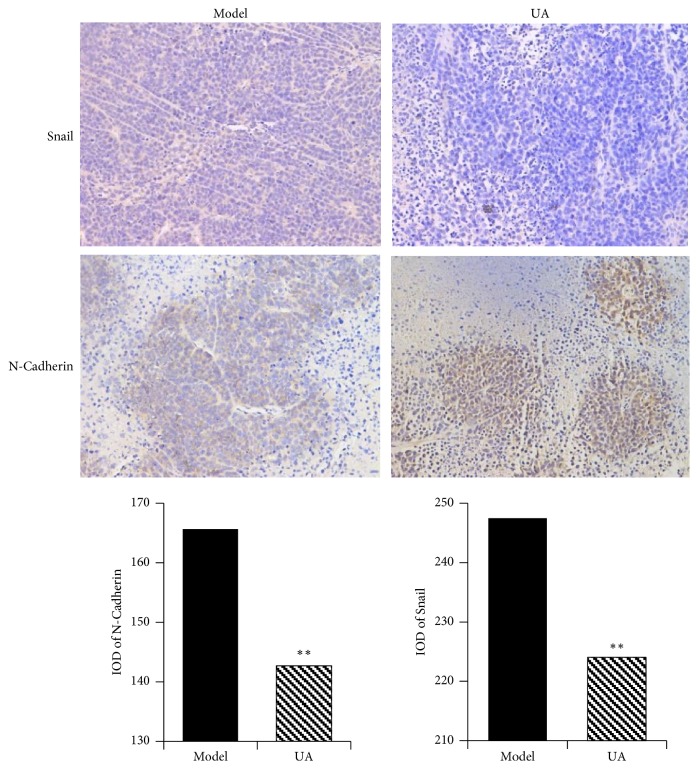
*UA inhibited the expression of EMT related proteins in mouse GC xenograft model.* Note: compared with model group, *∗∗*P<0.01.

Expressions of N-Cadherin and Snail in mouse GC xenograft model were examined by immunohistochemical analysis. As shown in Figure 7, compared with model group, the levels of N-Cadherin and Snail were significantly decreased in UA group (P<0.01).

### 3.6. Inhibitory Effects of UA on Axl/NF-*κ*B Pathway in Mouse GC Xenograft Model

**Figure 8:**
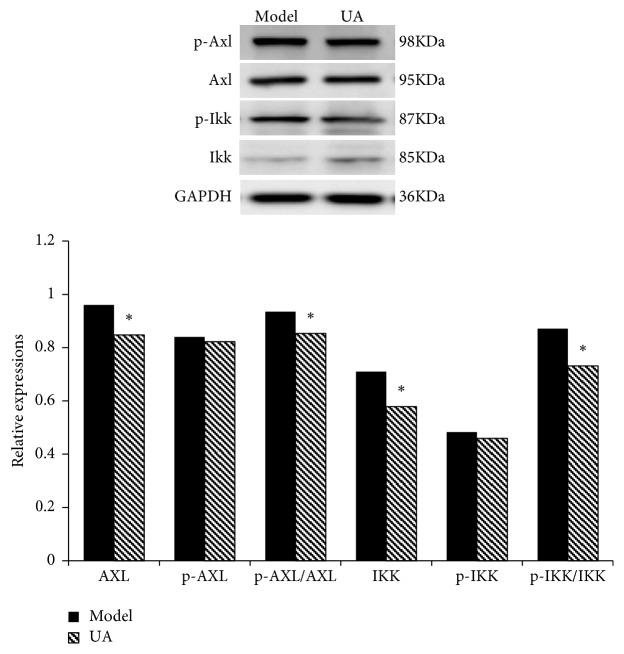
*Inhibitory effects of UA on Axl/NF-κB pathway in mouse GC xenograft model.* Note: compared with model group, *∗*P<0.05.

The key proteins in Axl/NF-*κ*B pathway were examined by Western blot in mouse GC xenograft model. As shown in Figure 8, compared with model group, UA downregulated the expressions of Axl, p-Axl, IKK*α*/*β*, and p-IKK*α*/*β*, and the values of p-Axl/Axl and p-IKK/IKK significantly decreased (P<0.05). These results indicate that the phosphorylations of Axl and IKK have been inhibited by UA.

## 4. Discussion

UA is a pentacyclic triterpene compound and exists in medicinal herbs such as* Oldenlandia diffusa *and *Radix actinidiae.* It has the effects of anti-inflammatory, antioxidant, and antitumor [10]. Studies show that UA inhibits growth of tumor cells through multiple functions, such as cytotoxicity [11], induction of apoptosis [12], and prevention of angiogenesis [13]. In this study, different concentrations of UA were applied to various GC cell lines, and its resistance effects were observed. The results show that UA distinctly inhibited the proliferation of BGC-823 and HGC-27 cells in dose-dependent manners. UA significantly inhibited the migration of BGC-823, AGS, and MGC-803 cells. UA induced apoptosis of BGC-823 cells. In conclusion, UA can inhibit cell proliferation and migration, while inducing cell apoptosis of GC cells. Results are consistent with previous reports [9, 10].

Metastasis is the leading cause of cancer deaths. Attenuation of intercellular adhesion and enhancement of cellular activity are the basis of tumor invasion and metastasis [14]. EMT plays a crucial role in the genesis, invasion, and metastasis of various tumors. In EMT process, epithelial cells are converted into mesenchymal cells, causing the loss of cell-cell adhesion and the cell polarity, by which means cells acquired migratory and invasive properties [15]. Studies have confirmed that EMT is closely related to the invasion and metastasis of GC [16]. During EMT, cadherin switches from E-cadherin to N-Cadherin; thus the upregulated expression of N-Cadherin is considered important EMT biomarkers [17]. Vimentin is a kind of mesenchymal protein that exists in mesenchymal cells. The activation of Vimentin regulates cytoskeletal protein and cell adhesion, which leads to the occurrence of EMT [18]. Snail, Slug, Smuc, ZEB, and Twist are the upstream transcription factors of EMT; they can competitively bind E-box sequences and cause epigenetic silence of E-cadherin gene, therefore promoting tumor metastasis [18]. In this study, we observed the effects of UA on the expressions of EMT related proteins. Results show that 25*μ*mol/L UA decreased the levels of N-Cadherin, Vimentin, Snail, and Twist in BGC-823 and MGC-803 cells. UA significantly reduced N-Cadherin and Snail expressions in mouse GC xenograft model. The results verified the inhibition effects of UA on EMT in GC from both* in vivo *and* in vitro*.

EMT is regulated by many signaling pathways. Axl is a member of receptor tyrosine kinase family and involves in the regulation of cell adhesion, proliferation, and cell transformation [19]. The overexpression of Axl can increase the invasiveness of GC [20]; the effect is related to induction of EMT [21, 22]. Breast cancer cells with overexpression of Axl have mesenchymal cellular phenotype; it does not rely on the existence of Axl's ligand Gas6. The application of Axl-shRNA or Axl inhibitor MP470 can inhibit Axl expression, cells transferred to epithelial-like phenotype, and the EMT phenotype reversed [23]. As a downstream transcription factor of Axl, the sustained activation of NF-*κ*B has the effect of promoting EMT [24]. Kiefel H et al. blocked Axl on the surface of breast cancer cells; the activity of NF-*κ*B pathway had decreased [25]. Huber et al. found that inhibition of NF-*κ*B could block EMT and affect epithelial plasticity and metastasis of breast cancer; on the contrary, activation of NF-*κ*B promotes EMT in breast cancer cells [26]. In the resting state of most cells, NF-*κ*B integrates with its inhibitor I*κ*B-*α*; under the activation of kinase IKK*α*, IKK*β*, and IKK*γ*, I*κ*B-*α* was phosphorylated and degraded, which expose the nuclear localization sequence of NF-*κ*B and promote the activity of EMT. Shishodia et al. found that UA can inhibit IKK*α* and p65 phosphorylation, leading to the suppression of NF-*κ*B activation induced by various carcinogens [27]. To further explore the mechanism of UA on EMT, we observed the effects of UA on expressions of key proteins in Axl/ NF-*κ*B pathway. Results show that 25*μ*mol/L UA decreased the levels of p-Axl, p-IKK*α*/*β*, and p-NF-*κ*B in BGC-823 cells, and it significantly decreased the p-Axl and p-IKK in mouse GC xenograft model. The results indicate that the inhibitory effects of UA on EMT may be associated with the regulation of Axl/NF-*κ*B signaling pathway.

In this study, 50*μ*mol/L UA damaged MGC-803 and AGS cells too much to proceed with subsequent experiments; further exploration of effective dose of UA is necessary.

## 5. Conclusion

UA can effectively inhibit the proliferation and migration and induce apoptosis of gastric cancer cells. The antitumor effect of UA is conducted by EMT inhibition, which may be associated with the regulation of Axl/NF-*κ*B signaling pathway.

## Data Availability

The images of this study are included within the article. The datasets used to support the findings of this study are available from the corresponding author upon request.

## Abbreviations

EMT: Epithelial-mesenchymal transition
UA: Ursolic acid
GC: Gastric carcinoma.
